# Adaptive Neuro-Fuzzy Inference System for Modelling the Effect of Slurry Impacts on PLA Material Processed by FDM

**DOI:** 10.3390/polym13010118

**Published:** 2020-12-30

**Authors:** Bahaa Saleh, Ibrahem Maher, Yasser Abdelrhman, Mahmoud Heshmat, Osama Abdelaal

**Affiliations:** 1 Mechanical Engineering Department, College of Engineering, Taif University, P.O. Box 11099, Taif 21944, Saudi Arabia; b.saleh@tu.edu.sa; 2 Department of Mechanical Engineering, Faculty of Engineering, Assiut University, Assiut 71515, Egypt; mheshmat@aun.edu.eg (M.H.); o.abdelaal@mu.edu.sa (O.A.); 3 Department of Mechanical Engineering, Faculty of Engineering, Kafrelsheikh University, Kafrelsheikh 33516, Egypt; ibrahemmaher@eng.kfs.edu.eg; 4 Department of Mechanical and Industrial Engineering, College of Engineering, Majmaah University, Al-Majmaah 11952, Saudi Arabia

**Keywords:** ANFIS, slurry impacts, polylactic acid, 3D printing, fused deposition modeling

## Abstract

In this research, the effect of water-silica slurry impacts on polylactic acid (PLA) processed by fused deposition modeling (FDM) is examined under different conditions with the assistance of an adaptive neuro-fuzzy interference system (ANFIS). Building orientation, layer thickness, and slurry impact angle are considered as the controllable variables. Weight gain resulting from water, net weight gain, and total weight gain are the predicting variables. Results uncover the accomplishment of the ANFIS model to appropriately appraise slurry erosion in correlation with comparing real data. Both experimental and ANFIS results are almost identical with average percentage error less than 5.45 × 10^−6^. We observed during the slurry impacts tests that all specimens showed an increase in their weights. This weight gain was finally interpreted to the synergetic effect of water absorption and the solid particles fragmentations immersed within the specimens due to the successive slurry impacts.

## 1. Introduction

In recent years, polymer materials have a growing interest in different industrial applications under conditions of slurry impacts, such as pipelines exposed to slurry stream in refinery and offshore petroleum industry [1,2,3,4,5], and sewage systems [6,7]. This growing attention to polymers is due to several factors: environmental requirements, cost reduction, corrosion in metallic counterparts, weight reduction, and electrical and thermal insulating properties [8]. The most common processing methods for polymers are molding and melt extrusion processes. Currently additive manufacturing (AM, also called 3D printing by the public), is a new manufacturing route for thermoplastic polymers such as PLA and ABS. Additive manufacturing comprising a group of techniques that sharing the common working principle of layer-by-layer manner to build the desired polymer, metal, ceramic, or composite object. Fused Deposition Modeling (FDM) is one of the additive manufacturing techniques for processing thermoplastics. In FDM, 3D polymer prototypes and objects are built based its Computer-Aided Design (CAD) data. The process starts with a filament which is heated and deposits successively by a nozzle on a printing bed.

PLA is one of the most used material for FDM since it is used for different industrial applications. Moreover, PLA is renewable, compostable, biocompatible, and has good damping properties and low cracking rate [9,10]. Furthermore, PLA has a potential in slurry applications such as exterior automotive applications [11], the construction industry [11], water pump impeller [12,13], water filters [14], pipes and fittings [15], and antenna radomes [16]. Despite the clear understanding of slurry erosion behavior of metallic materials due to slurry impacts, the current knowledge on the effect of slurry impacts on polymer material, especially additively manufactured polymers is limited. Therefore, it is crucial to strengthen the literature theoretically and experimentally with predictive and reliable information on the erosion behavior of printed polymers for a given engineering design or application. 

In this regard, developing intelligent systems and soft computing models for prediction of the effect of slurry impacts on additively manufactured polymers has drawn considerable research attention. Soft computing techniques are useful to provide mathematical relations when exact relations are not available. These techniques differ from conventional computing methods in that they tolerate imprecision, uncertainty, partial truth, approximation, and metaheuristics [17]. Two famous forms of artificial intelligence; including neural networks and fuzzy systems, are used together to enhance the developed models of 3D printed PLA [17]. Two famous forms of artificial intelligence; including neural networks and fuzzy, were used together to enhance the developed models of 3D printed polylactic acid. 

An adaptive neuro-fuzzy inference system (ANFIS) is one of the soft computing techniques which playing a great role in modeling accurate input–output matrix relationships [18]. ANFIS is ideal to predict weight gain based on input variables due to the nonlinear condition in the 3D printed PLA process. Additionally, ANFIS has been used in modeling and evaluating slurry erosion of metallic materials. For example, Hassan et al. [19], developed a fuzzy model to evaluate and predict the slurry erosion of 5127 steel. Their developed model achieved a good agreement with experimental results. The slurry erosive wear behavior of Al6061 alloy was predicted using fuzzy logic approach by Ramesh et al. [20], and it was found that the predicted values were in close agreement with the experimental results. An ANFIS model was proposed for estimation of erosion rate of copper particles flow through an aluminum 3003 alloy elbow were developed by Shamshirband et al. [21]. They reported that ANFIS model achieved high reliability in forecasting maximum and total erosion rates. In the context of FDM, ANFIS has been used recently to predict different printing output based on printing parameters. For example, Dambatta et al. [22] used the layer thickness, orientation angle and structural geometry to predict the volumetric shrinkage of FDMed products. Rajpurohit and Dave [23] used ANFIS to anticipate the tensile strength of PLA printed parts based on the raster angle, layer height, and raster width. Yadav et al. [24] studied the effects of extrusion temperature, layer height, and material density of FDMed products on the tensile strength of materials like PETG, ABS and multi-material (60% ABS + 40% PETG). They used ANFIS to estimate the maximum tensile strength of these printed products. 

To the best of our knowledge, no significant investigation has been directed to understand the behavior of FDM processed PLA parts subjected to slurry impacts. To fill this gap, the present study focusses on developing an ANFIS model to predict the output of slurry impact of FDM-processed PLA products. The addressed printing parameters are layer thickness, building orientation in addition to the impact angle induced by the slurry particles on the printed products target surface.

## 2. Materials and Methods

### 2.1. 3D Printing Methodology and Parameters

Taguchi’s L9 orthogonal array is used for planning the experiments based on parameters affecting both the FDM processing and slurry impact as well as the levels at which they varied, as shown in Table 1. Build orientation and layer thickness are the critical parameters affecting the FDM process, and the impact angle parameter is a key factor connected with the slurry impact condition. The input parameters and their levels are considered according to the literature survey [25,26,27,28]. 

**Figure 1:**
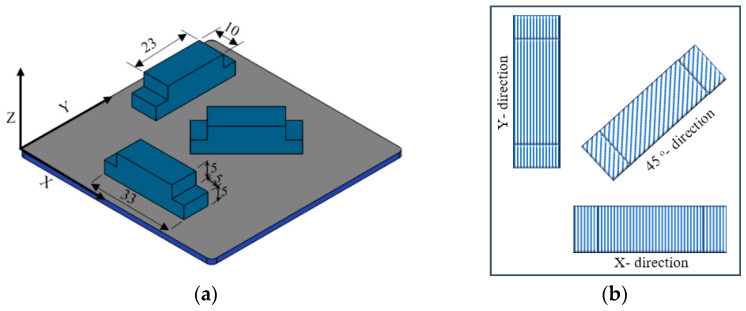
Test sample model: (**a**) Geometrical details and building orientation of the sample on the FDM building platform; (**b**) one direction linear infill hatching pattern.

A Robota Pro+2^®^ FDM 3D-printer (ROBOTA, Alexandria, Egypt) was used to fabricate the PLA samples with the dimensions as shown in Figure 1 in X-, Y-, and 45°- build orientations and 0.1, 0.2-, and 0.3-layer thicknesses. The PLA filament was obtained from eSUN, Inc. (Shenzhen, China) with properties shown in Table 2. The 3D CAD model of samples was created using Solidworks^®^ CAD software (Dassault Systèmes, Waltham, MA, USA), and then converted into STL (stereolithiography) file format. The STL file was then exported to Cura^®^ software package (Ultimaker B.V., Utrecht, Netherlands) in which processing parameters were set, the model was sliced, and G-code was generated and sent to the FDM machine for manufacturing. All the specimens were built with a one direction linear infill pattern, 90° raster angle, 100% infill density, 50 mm/sec print speed, 200 °C extrusion temperature, 60 °C hot bed temperature.

### 2.2. Slurry Impact Experimental Setup

**Figure 2:**
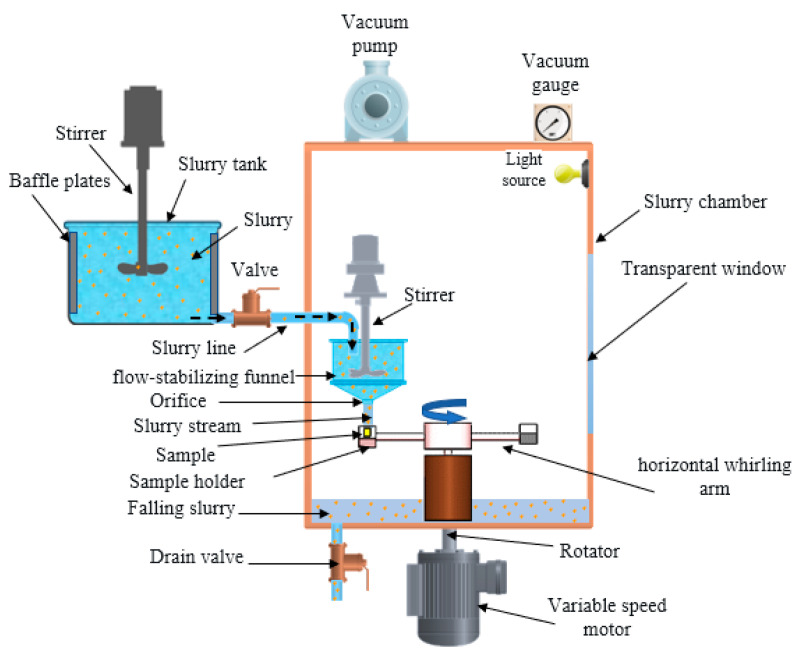
The whirling arm slurry erosion test (WASET) rig [30,31].

**Figure 3:**
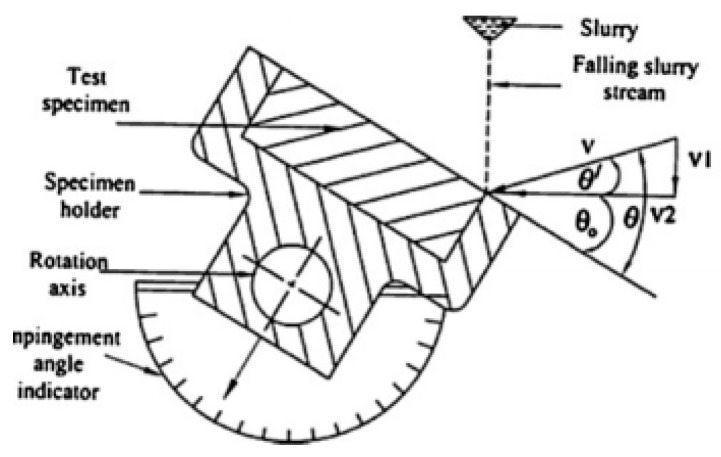
A schematic diagram of the impact angle and impact velocity of the test [32].

The slurry impact tests were conducted on the whirling arm slurry erosion tester (WASET) rig (Assiut university, Assiut, Egypt) on which the sand particles are driven and accelerated by gravity fallen water. As shown in Figure 2, the WASET rig is comprised of fresh slurry mixture unit (25 L capacity tank, stirrer, flow control valve, pipe), slurry chamber (funnel, stirrer, vacuum pump, vacuum gauge), and sample mounting and rotation unit (motor, speed controller, rotor, two horizontal whirling arms, two sample holders, and impact angle fixture (0–90°)). Figure 3 shows a schematic diagram of the impact angle and impact velocity of the test. The details of operation and performance has been described elsewhere [29,30].

The predetermined amounts of fresh water and sand are added and mixed homogeneously in the mixing tank, then the slurry fed into the gravity flow-stabilizing funnel through pipe then passing through the funnel orifice and impacting the as-printed samples target surfaces (23 mm × 10 mm) with desired flowrate. Testing conditions for slurry erosion of FDMed PLA are giving in Table 3. After each test run, samples were cleaned with hot air jet and soft brush. The amount of mass loss/gain was estimated by weighting the sample before and after the test run using a digital electronic balance (Sartorius A200, Goettingen, Germany) with an accuracy of ±0.1 mg. After testing, slurry mixture was drained out.

### 2.3. ANFIS Model

Each intelligent strategy has specific computational properties that make them appropriate for specific issues. For instance, while neural networks are acceptable at perceiving patterns, they are bad at clarifying how they get at their decisions. Fuzzy logic frameworks, which can dissuade uncertain data, are acceptable at clarifying their decisions yet they cannot consequently procure the rules they use to settle on those decisions. These impediments have been a focal main impetus behind the production of keen hybrid frameworks where at least two methods are consolidated in a way that conquers the constraints of individual procedures.

To empower a framework of managing cognitive uncertainty in a way more like humans, one may consolidate the idea of fuzzy logic into the neural networks. The computational cycle imagined for fuzzy-neural systems (ANFIS) is as per the following. It begins with the advancement of a fuzzy-neuron dependent on the comprehension of biological neuronal morphologies, trailed by learning components. This prompts the accompanying three stages in a fuzzy-neural computational process:

1—Advancement of fuzzy-neural system driven by biological neurons,

2—System of synaptic connections which consolidates fuzziness into neural network,

3—Advancement of learning calculations.

Neural networks are utilized to tune membership function of fuzzy frameworks that are utilized as decision-making frameworks. Albeit fuzzy logic can encode master information legitimately utilizing rules with linguistic labels, it typically takes a great deal of effort to plan and tune the membership functions which quantitatively characterize these linguistic labels. Neural networks learning strategies can mechanize this cycle and generously lessen the improvement time and cost while improving execution [33].

**Figure 4:**
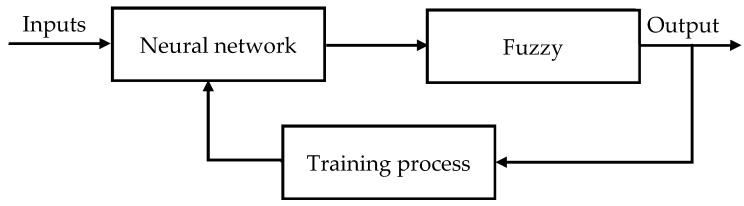
Fuzzy neural system model.

The multi-layered neural network drives the fuzzy logic inference system appeared in Figure 4 is utilized to fabricate the ANFIS model. ANFIS is a fuzzy inference system executed in the framework of an adaptive neural network. ANFIS can be utilized to build an input-output planning dependent on human information as fuzzy if-then rules just as foreordained input-output data sets for neural network preparing. The membership function boundaries are registered by the ANFIS demonstrating to follow the known trial input-output data.

**Figure 5:**
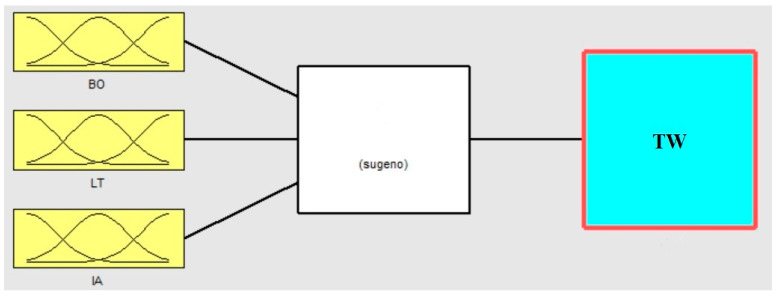
Fuzzy role architecture of the generalized bell membership function.

ANFIS was built in MATLAB software (MathWorks, Inc, Natick, MA, USA) and different membership functions were used to train ANFIS. Three membership functions of building orientation, layer thickness, and impact angle were chosen to create the ANFIS model as shown in Figure 5. The generalized bell membership function gives the lowermost training error of total weight gain, so it was implemented for the ANFIS training method in this work. The fuzzy rule construction of ANFIS when generalized bell membership function is adopted based on the Sugeno fuzzy model as shown in Figure 5.

**Figure 6:**
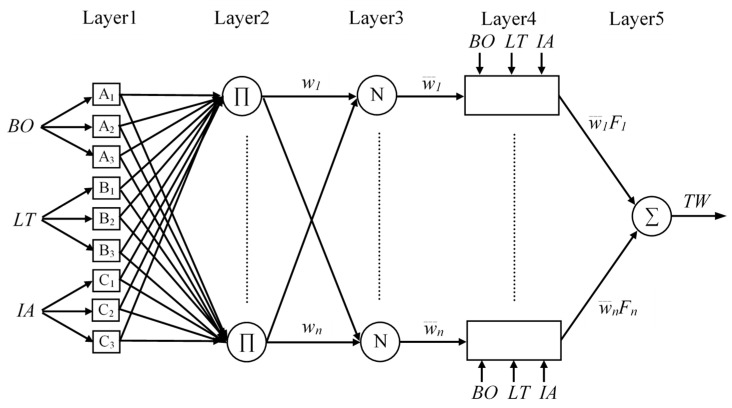
ANFIS architecture for three input Sugeno fuzzy model.

ANFIS uses five network layers to achieve the following fuzzy interpretation steps as shown in Figure 6. Where layer (1) is the input parameters, (2) is the fuzzy set database layer, (3) is the fuzzy rule base structure layer, (4) is the decision making layer, and (5) is the output defuzzification layer; more information are available in literature [34,35].

**Figure 7:**
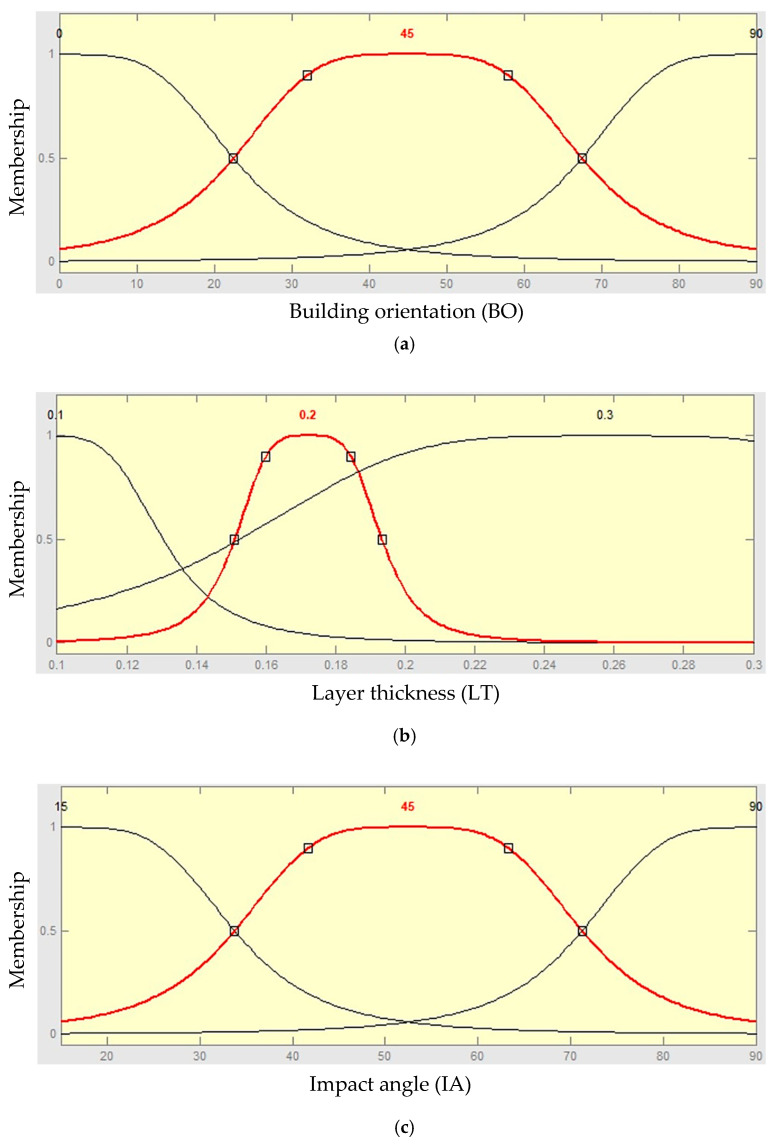
Membership functions of input parameters after training: (**a**) membership function of building orientation (BO), (**b**) membership function of layer thickness (LT), and (**c**) membership function of impact angle (IA).

The five layers are described as follows:

Layer 1: the output of this layer is the step to which the given input satisfies the linguistic label associated to this node. Generalized bell shape membership functions (*gbellmf*) are generally used to represent the verbal terms as the connection between the input parameters and output response is not linear, as shown in Figure 7.

First parameter membership functions:

(1) $$A_{i}\left(BO\right)=\frac{1}{1+{\left|\frac{BO-c_{i1}}{a_{i1}}\right|}^{2b_{i1}}}$$

Second parameter membership functions:

(2) $$B_{i}\left(LT\right)=\frac{1}{1+{\left|\frac{LT-c_{i2}}{a_{i2}}\right|}^{2b_{i2}}}$$

Third parameter membership functions:

(3) $$C_{i}\left(IA\right)=\frac{1}{1+{\left|\frac{IA-c_{i3}}{a_{i3}}\right|}^{2b_{i3}}}$$

where {*a_i_*_1_, *a_i_*_2_, *a_i_*_3_, *b_i_*_1_, *b_i2_, b_i_*_3_, *c_i_*_1_, *c_i_*_2_, *c_i_*_3_} are the parameter set.

As the values of these limits’ modification, the functions shapes vary consequently as shown in Figure 7, thus displaying several forms of membership functions on linguistic tags *Ai*, *Bi* and *Ci*. Parameters in this layer are denoted to as attitude parameters.

Layer 2: The firing strength of the connected rule can be computed by every node. The outputs are:

Top neuron:

*w*_1_ = *A*_1_(*BO*) × *B*_1_(*LT*) × *C*_1_(*IA*) (4)

Bottom neuron: *w_n_* = *A*_3_(*BO*) × *B*_3_(*LT*) × *C*_3_(*IA)*

Layer 3: every node in this layer is considered by N to indicate the regulation of the firing levels. The output of top and bottom neuron is normalized as follow:

Top neuron:

(5) $${\bar{w}}_{1}=\frac{w_{1}}{w_{1}+w_{2}+\ldots +w_{n}}$$

Bottom neuron: ${\bar{w}}_{n}=\frac{w_{n}}{w_{1}+w_{2}+\ldots +w_{n}}$

Layer 4: the output in this layer is the result of the normalized level of firing and the individual output of the main rule and last rule.

Top neuron:

(6) $${\bar{w}}_{1}F_{1}\;=\;{\bar{w}}_{1}\;(a_{1}BO\;+\;b_{1}LT\;+\;c_{1}IA)\;$$

Bottom neuron: $\;{\bar{w}}_{n}$*F_n_* = ${\bar{w}}_{n}$ (*a_n_BO* + *b_n_LT* + *c_n_IA*)

Layer 5: the node in this layer figures the general framework as the amount of every input, i.e.,:

(7) $$TW=\;{\bar{w}}_{1}F_{1}\;+\;{\bar{w}}_{2}F_{2}\;+\;...\;+\;{\bar{w}}_{n}F_{n}$$

**Figure 8:**
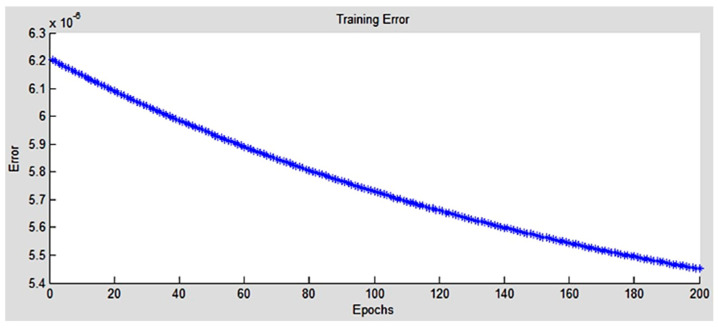
Training error.

In the event that a training set {(x^k^, y^k^), k = 1, ……, K} was given, the boundaries of the hybrid neural network (which decide the state of the membership functions of the premises) can be realized by descent-type strategies. The error for sample k can be calculated by:

E_k_ = (y^k^ − o^k^)^2^ (8)

where y^k^ is the required output and o^k^ is the modeled one by the hybrid neural network. Throughout training, the output data set were utilized to perform 200 rounds of learning with an average error of 5.45 × 10^−6^ as shown in Figure 8.

## 3. Results and Discussion

### 3.1. Weight Gain of 3D PLA due to Slurry Impacts

**Figure 9:**
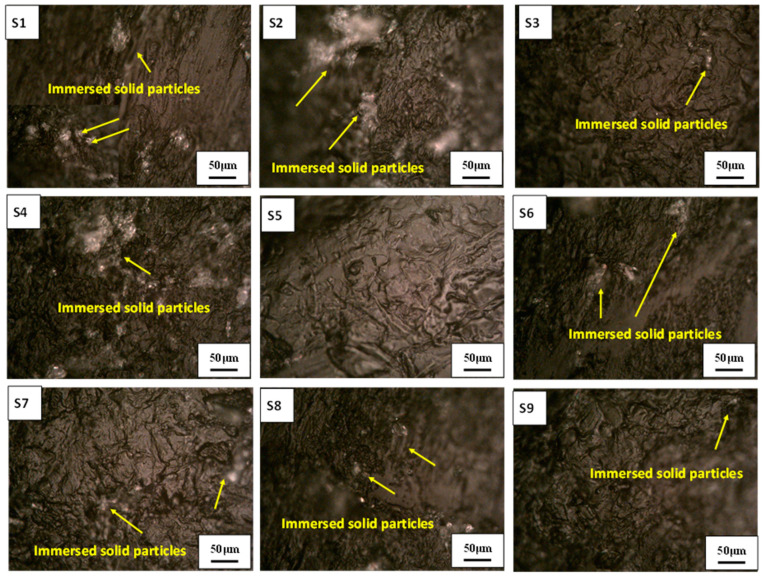
Optical microscopy of the FDMed PLA specimens after subjecting them to the slurry impacts. S1 (BO: Y, LT: 0.1, IA: 15°), S2 (BO: Y, LT: 0.2, IA: 45°), S3 (BO: Y, LT: 0.3, IA: 90°), S4 (BO: 45°, LT: 0.1, IA: 45°), S5 (BO: 45°, LT: 0.2, IA: 90°), S6 (BO: 45°, LT: 0.3, IA: 15°), S7 (BO: X, LT: 0.1, IA: 90°), S8 (BO: X, LT: 0.2, IA: 15°), and S9 (BO: X, LT: 0.3, IA: 45°).

**Figure 10:**
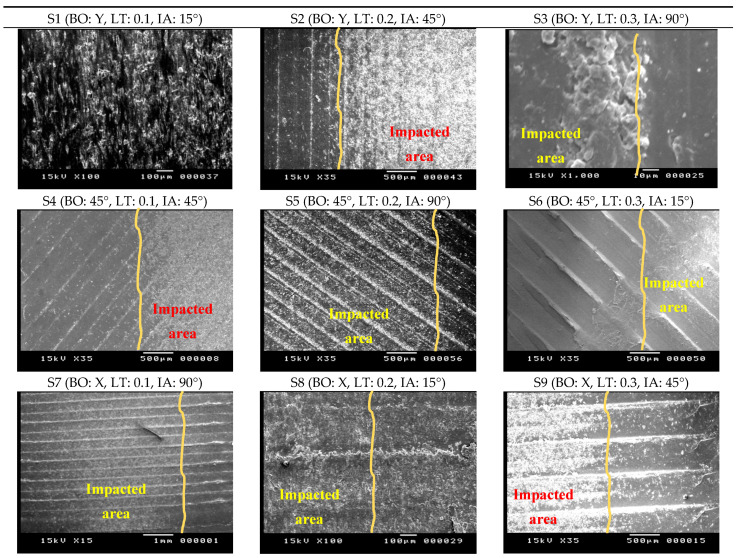
SEM images of the 3D printed PLA specimens after subjecting to the slurry impacts.

**Figure 11:**
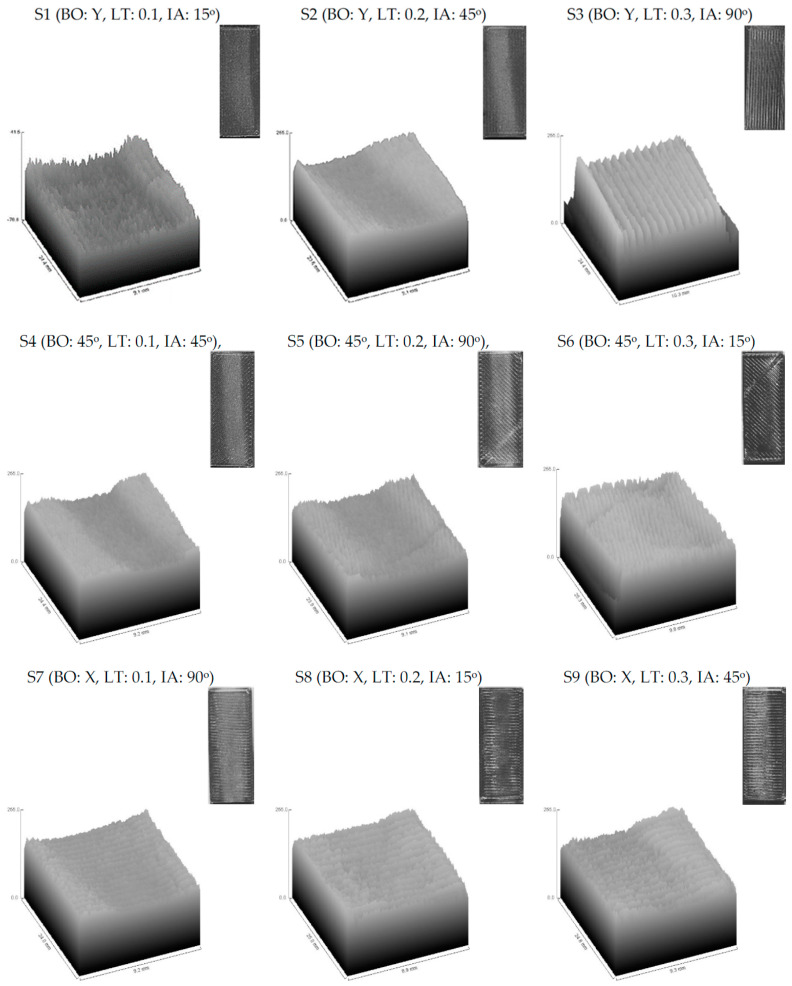
Representative 3D surface plots of surface topography of FDMed PLA specimens after subjecting to the slurry impacts.

From previous work by authors [36] the manufactured specimens at different building conditions (Building orientation: BO, and layer thickness: LT) were tested under different slurry impact conditions (Impact angle: IA). The slurry impacts on the specimens caused weight gain for all the examined specimens at all building and testing conditions. The observed weight gain of the specimens was postulated to two reasons: (a) water absorption, and (b) the embedded sand particles in the surfaces of the specimens [36]. Figure 9 and Figure 10 show the impacted surface of the specimens scanned using an optical microscope (OM) and SEM, respectively. The OM images illustrate one of the reasons of the weight gain of the specimens after the slurry impact tests, i.e., the immersed fragmentation of the solid particles in the top surface of the specimens after the slurry impact test. Figure 10 illustrates the boarders between the impacted and unimpacted areas using SEM images. The first region is subjected to slurry impact and the other one is not. The areas that are subjected to slurry impacts are shown in white color. The peaks of the beads are almost smashed, and the surface of these areas is almost homogeneous without any severe hills or bottoms, whereas the darker areas are the original surface after the 3D printing process where the hills and bottoms of the printed beads are clear and the difference between them is high. This is also clear from the 3D surface profile images shown in Figure 11 extracted from the images of each sample after testing using the image analysis software (ImageJ, National Institutes of Health, USA) for a quantitative comparison of surface topography. Figure 11 also reveals that in specimens which have the highest LT (i.e., 0.3 mm in S3, S6, S9), the plastic deformation of these specimens in the impacted area is limited and, hence, resulted in higher surface roughness.

**Figure 12:**
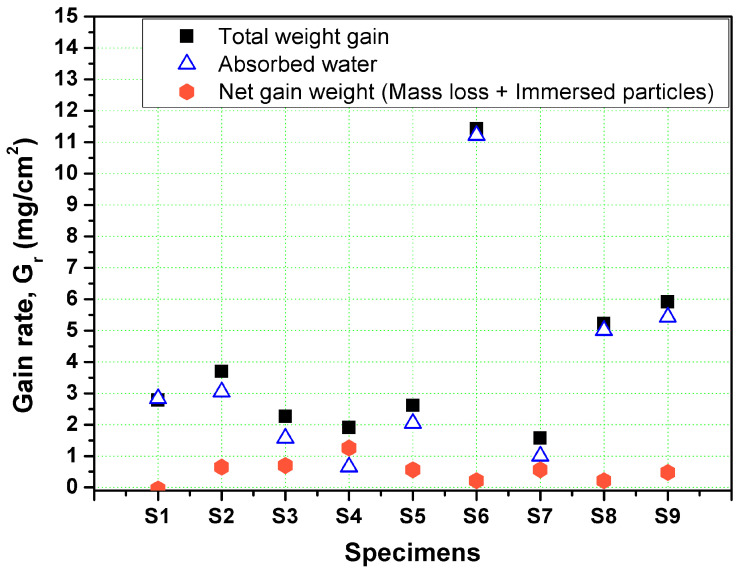
Gain rate in the weight of the specimens, absorbed water, and the net gain after impacting by 112.37 g of solid particles.

Figure 12 shows the gain rate, Gr (mg/cm^2^), in the weight of each specimens after impacting by 112.37 g of SiO_2_ solid particles with 427.5 µm average size. The gain rate, Gr, was determined by dividing the absolute weight gain (mg) of the specimen by the eroded surface area. The area of the eroded surface, EA, represents nearly the half of the specimen top surface area, (i.e., 5 mm × 23 mm). In addition, weights of the absorbed water due to the slurry impact tests were determined by drying the specimens after tests, and the weight of the absorbed water for each specimen was determined by determining the difference between the weights before and after the drying process, as shown in Figure 9. Finally, the difference between the total gain weight and the weight of the absorbed water for each specimen was determined to represent the net gain weight of the specimen after the slurry impact test. The net gain weight is due to the penetrations and immersions of the solid particles accompanied with the mass loss which may be occurred due to the successive slurry impacts, as shown in Figure 9.

The highest total weight gain is observed at specimens S6 (BO: 45°, LT: 0.3 mm, and IA: 15°) and specimen S9 (BO: X-direction, LT: 0.3 mm, and IA: 45°). In contrast, the lowest weight gain is observed at specimens S7 (BO: X-direction, LT: 0.1 mm, and IA: 90°) and specimen S4 (BO: 45°-direction, LT: 0.1 mm, IA: 45°).

The impact angle (IA) and the layer thickness (LT) are the significant parameters affecting the water gain, by increasing the LT the amount of the absorbed water during testing time is increased. While the IA only is the main factor influencing the amount of the embedded solid particles in the top surface of the impacted specimens. By decreasing the IA, the amount of the immersed particles in the top surfaces of the specimens is increased. At normal impact angles (IA = 90°), the impacted particles penetrate inside the top surface of the specimen for a small distance without any shear, as the horizontal force of the impacting force equals to zero at normal angles. Then, due to the successive impacts of the new particles, the old-immersed particle will be removed, or it will be fractured and leaving behind a small fragmentation immersed inside the top surface of the specimen. However, at small IA, the high shearing force (horizontal component of the impacting force) and the formed chips in front of the impacted particle will let the adhesion force between the immersed particle and the specimen is very strong and the successive impacts will hardly remove this particle and if there are some fragmentations due to the impacting process, their sizes will be larger and, in turn, their weight gain will be increased due to this process.

### 3.2. ANFIS Model Analysis

**Figure 13:**
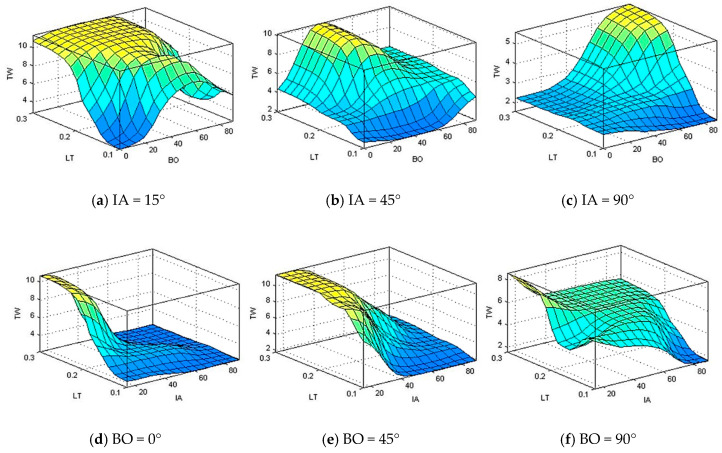
Total weight gain (TW) in relation to change of building orientation (BO) and layer thickness (LT), and impact angle (IA) at (**a**) IA = 15°, (**b**) IA = 45°, (**c**) IA = 90°, (**d**) BO = 0°, (**e**) BO = 45°, and (**f**) BO = 90°.

**Figure 14:**
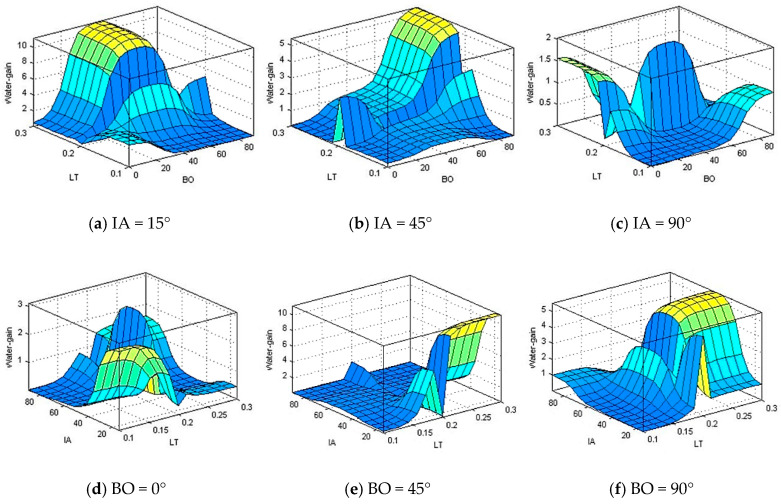
Water gain in relation to change of building orientation (BO), layer thickness (LT), and impact angle (IA) at (**a**) IA = 15°, (**b**) IA = 45°, (**c**) IA = 90°, (**d**) BO = 0°, (**e**) BO = 45°, and (**f**) BO = 90°.

**Figure 15:**
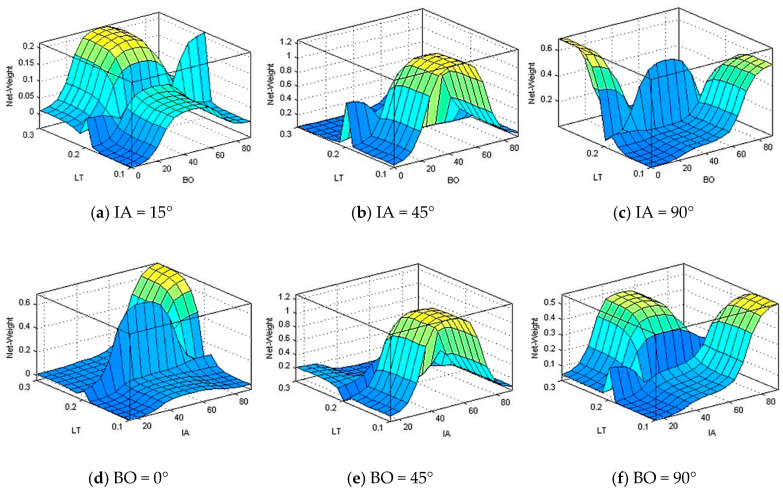
Net weight gain in relation to change of building orientation (BO), layer thickness (LT), and impact angle (IA) at (**a**) IA = 15°, (**b**) IA = 45°, (**c**) IA = 90°, (**d**) BO = 0°, (**e**) BO = 45°, and (**f**) BO = 90°.

Using the developed ANFIS model, the weight gain variables of PLA material were acquired from the three input variables. The combined effect of the three variables on the output variation of slurry erosion can be shown in Figure 13, Figure 14 and Figure 15. Figure 13 shows the surface plots that explain the effects of the water silica slurry impacts parameters on total weight gain.

According to Figure 13, impact angle (IA) has great effect followed by layer thickness (LT) on total weight gain, while building orientation (BO) has a minor effect on total weight gain. Similar kinds of observations have been obtained experimentally. Moreover, at low level of impact angle, the layer thickness has a considerable effect on weight gain. The total weight gain increases with increasing the layer thickness during all range of layer thickness. Also, at low and medium levels of impact angles, the low and high levels of building orientation do not have a considerable effect on weight gain. Additionally, at high level of impact angles and layer thickness, the total weight gain increases with the increase of building orientation.

According to Figure 14 and Figure 15, there is no clear pattern or relation between the input parameters (impact angle, layer thickness, and building orientation) and output parameters (water gain and net weight gain). Figure 14 shows the effects of input variables on water gain. It can be observed from Figure 14 that, the maximum water gain takes place at low level of impact angel, medium level of building orientation, and high level of layer thickness.

Figure 15 shows the surface plots of the water silica slurry impacts parameters effect on net weight gain. It can be seen from Figure 15 that, the maximum net weight gain around 0.6 and appears at low building orientation and high layer thickness along with a high impact angle.

## 4. Conclusions

In the present study, the effect of slurry impacts on PLA material manufactured by FDM 3D printing technique was predicted under several building and testing conditions using ANFIS. The obtained results from the presented ANFIS model were compared with previously obtained experimental results and found to be almost identical. The three studied factors are building orientation (BO), layer thickness (LT), and impact angle of the slurry (IA). For all specimens, the weight gain is the main tendency after subjecting to the water-sand slurry impact. The water absorption and the embedded particles/fragments are the main reasons behind that weight gain. The results reveal that the developed ANFIS model can be effectively adopted to model and predict the effect of slurry impacts on PLA material processed by FDM. The precise output values ensure the effective use of the developed ANFIS model.

## Figures and Tables

**Table 1:** Taguchi’s L9 array of parameters and their levels considered for the experimentation.

Parameter	Name	Minimum	Middle	Maximum
BO	Build orientation	X	45	Y
LT	Layer Thickness (mm)	0.1	0.2	0.3
IA	Impact Angle (degree)	15	45	90

**Table 2:** PLA’s properties (supplier’s data).

Property	Value
Density (g/cm^3^)	1.24
Printing temperature (°C)	190–210
Tensile strength (MPa)	65
Distortion Temp (°C, 0.45 MPa)	56
Melt Flow Index (g/10 min)	5 (190 °C/2.16 kg)
Elongation at break (%)	8

**Table 3:** Testing conditions for slurry impact of FDM-processed PLA.

Parameter	Value
Testing velocity	15 m/s
Impingement angles	15°, 45° and 90°
Orifice diameter	3 mm
Orifice to sample distance	40 mm
Erodent	SiO_2_
Particle size	355~500 µm
Concentration	1% wt.
Temperature	25 °C
Material	FDMed PLA

## Data Availability

Not applicable.
